# Acute Hypoxic Exposure May Contribute to Depressive-Like Behaviors in Mice Through Disruption of Gut Microbiota Homeostasis

**DOI:** 10.31083/AP47626

**Published:** 2026-04-20

**Authors:** Ruiying Cheng, Yajun Qiao, Huimin Zheng, Xiaohui Li, Qiannan Wang, Xingfang Zhang, Jianv Wang, Lixin Wei, Tingting Gao, Hongtao Bi

**Affiliations:** ^1^Qinghai Provincial Key Laboratory of Tibetan Medicine Pharmacology and Safety Evaluation, Northwest Institute of Plateau Biology, Chinese Academy of Science, 810008 Xining, Qinghai, China; ^2^School of Psychology, Chengdu Medical College, 610500 Chengdu, Sichuan, China; ^3^University of Chinese Academy of Sciences, 10049 Beijing, China; ^4^Department of Psychiatry, The People's Hospital of Jiangmen, Southern Medical University, 529000 Jiangmen, Guangdong, China; ^5^Medical College, Qinghai Minzu University, 810001 Xining, Qinghai, China; ^6^Medical College, Qinghai University, 810001 Xining, Qinghai, China; ^7^Department of Pharmacy, Xijing Hospital, Fourth Military Medical University, 710032 Xi'an, Shaanxi, China

**Keywords:** high altitude, acute hypoxia, depression, gut–brain axis, gut microbiota, tryptophan metabolism

## Abstract

**Background:**

Depression represents a major global disease burden. High-altitude hypoxia is closely associated with an increased incidence of depressive symptoms; however, the underlying mechanisms remain unclear.

**Methods:**

In this study, mice were subjected to 7 days of hypoxic exposure simulating high-altitude environments at 3000 m (14.4% O_2_) and 4000 m (12.7% O_2_). Depressive-like behaviors were assessed using the open field test, tail suspension test, and forced swim test. Enzyme-linked immunosorbent assays were employed to measure markers related to inflammatory responses, oxidative stress, and hypothalamic–pituitary–adrenal (HPA) axis activity. Additionally, 16S ribosomal RNA sequencing was performed to analyze alterations in the gut microbiota. Untargeted metabolomics was used to examine metabolite changes in the colon and hippocampus. Validation analyses included measurements of hippocampal immunofluorescence density and cortical neurotransmitter levels.

**Results:**

Hypoxic exposure induced depressive-like behaviors, as well as colonic and hippocampal tissue damage. It also exacerbated inflammation and oxidative stress, reduced gut microbiota diversity, disrupted tryptophan metabolism, decreased cortical neurotransmitter levels and brain-derived neurotrophic factor (BDNF), and increased the immunofluorescence density of hippocampal neuron–glial antigen 2 and oligodendrocyte transcription factor 2. These effects exhibited an intensity–toxicity relationship.

**Conclusions:**

Acute hypoxia is associated with gut microbiota imbalance, disrupted tryptophan metabolism, inflammatory responses, and dysfunction of the HPA axis, alongside negative emotional states. These multidimensional alterations are strongly correlated, suggesting a potential regulatory network involving the gut microbiota–tryptophan metabolism axis in hypoxia-induced emotional changes.

## Main Points

• Seven days of simulated high-altitude hypoxia (3000 m, 4000 m) induces depressive-like behaviors in mice.

• Hypoxia at 4000 m (12.7% O_2_) causes more severe depressive symptoms, gut dysbiosis, metabolic disruption, and tissue damage than hypoxia at 3000 m (14.4% O_2_).

• An imbalance in the gut microbiota initiates the response: hypoxia increases the abundance of proinflammatory bacteria (e.g., *Staphylococcaceae* and *Corynebacteriaceae*) and reduces the abundance of short-chain fatty acid (SCFA)-producing taxa (e.g., *Lachnospiraceae* and *Ruminococcaceae*), disrupting colonic and hippocampal tryptophan metabolism.

• This axis drives a pathological cascade—metabolic changes trigger inflammation, oxidative stress, hypothalamic–pituitary–adrenal (HPA) axis dysfunction, and hippocampal damage and lower levels of gamma-aminobutyric acid (GABA), brain-derived neurotrophic factor (BDNF), 5-hydroxytryptamine (5-HT), and norepinephrine (NE)—leading to depressive-like outcomes.

• This study identifies gut microbiota and tryptophan metabolism as key targets for assessing and treating depression in high-altitude populations.

## 1. Introduction

Depression is a common mental disorder. Owing to its high disability rate, it ranked among the top 25 contributors to the global disease burden in 2019 [1]. The Coronavirus Disease 2019 (COVID-19) pandemic impacted mental health, further worsening the disease burden linked to depression. Thus, mitigating the global health burden and addressing mental health challenges are essential [2]. Depression onset is shaped by multiple factors, including genetics, social environments, and geographical location [3]. Among these factors, high-altitude hypoxia has attracted considerable attention as a natural environmental factor. As global climate change has altered high-altitude ecosystems and human activities (e.g., mountaineering, plateau development, and military deployments) in these regions have expanded, more than 80 million permanent residents and 35 million annual visitors worldwide are now exposed to hypoxic conditions (≥2500 m altitude), creating a pressing need to understand the toxicological link between hypoxia and mental health [4, 5]. Epidemiological evidence has consistently linked higher residential altitude to increased depressive symptom scores and suicide rates. A landmark meta-analysis revealed that the pooled prevalence of depressive symptoms in high-altitude populations is 17.9%, which is higher than that reported in low-altitude regions such as China (1.8%), Brazil (14%), and 27 European countries (6.38%) [6, 7, 8]. Clinically, this association is even more concerning: patients with major depressive disorder (MDD) who travel to high altitudes often experience rapid exacerbation of core symptoms, with some cases progressing to treatment-resistant depression, a phenomenon linked to prolonged exposure to hypoxic conditions [9]. These observations frame high-altitude hypoxia not only as a physiological challenge but also as an environmental stressor capable of inducing neurotoxic effects via cascading disruptions to biological systems.

High-altitude environments involve multiple concurrent stressors (e.g., hypoxia, low temperature, and intense radiation), with hypoxia being the primary driver of depression [10]. From a toxicological standpoint, hypoxia exerts multitarget toxicity—inducing immune dysregulation, hormonal imbalance, and intestinal/brain tissue damage—but the direct biological mediators linking hypoxia to depressive traits remain poorly defined. In recent years, the gut‒brain axis (a key bridge between the gut microbial community and the central nervous system (CNS)) has emerged as a critical framework for understanding the impact of hypoxia on depression; this axis thus provides a core entry point for dissecting hypoxia‒depression connections. The gut‒brain axis is a bidirectional communication network that links gut microbes to the CNS via microbial metabolites, immune signaling, and the vagus nerve [11, 12, 13]. Notably, the gut microbial composition and metabolic features differ sharply between long-term residents of high-altitude hypoxic regions and those in low-altitude areas [14]. Acute hypoxic exposure disrupts the gut microbial balance [15]; this dysbiosis impairs the intestinal barrier, triggering systemic inflammatory responses that affect the brain—with 7 days of acute hypoxia alone sufficient to cause significant intestinal mucosal injury [16]. Gut microbial dysbiosis also reduces short-chain fatty acid (SCFA) levels, which amplifies hypothalamic‒pituitary‒adrenal (HPA) axis overactivity and hinders neurotransmitter synthesis, ultimately promoting depression [17]. While fecal microbiota transplantation (FMT) can alleviate oxidative stress injury in the hippocampus caused by acute hypoxia [15], critical gaps persist in the understanding of the toxicology of this pathway. Most studies have focused on chronic hypoxia, and the intensity‒toxicity relationship between acute hypoxia levels and depressive-like effects remains uncharacterized. Additionally, existing studies have typically examined individual gut‒brain axis components rather than integrating them into a stress‒toxicity cascade; furthermore, the key microbial taxa and metabolic factors linking gut dysbiosis to hippocampal damage remain unvalidated.

To address these existing research gaps, we designed a study to elucidate the toxicological cascade through which acute hypoxia may contribute to depressive-like phenotypes in mice. This study aimed to investigate how hypoxic stress disrupts the gut microbial ecology and may contribute to a series of cascades that ultimately lead to neurotoxic pathways, which in turn trigger depressive-like behaviors in mice and link gut perturbations to hippocampal damage, through the validation of downstream toxic effects. This research provides a toxicological basis for the risk assessment of high-altitude hypoxic exposure and identifies intervention targets to alleviate its neuropsychiatric toxicity and ultimately reduce the health burden of depression in high-altitude ecosystems.

## 2. Materials and Methods

### 2.1 Experimental Animals

Thirty 5- to 6-week-old male Kunming (KM) mice were procured from Sipeifu Biotechnology Co., Ltd (Beijing, China). The mice were housed at ambient temperatures of 22–25 °C, with a 12-h light/dark cycle and unrestricted access to standard rodent feed and water.

### 2.2 Experimental Groups and Treatment

After two weeks of adaptive feeding, 30 mice were randomly divided into three groups: the control (21% O_2_), hypoxia 3000 (HYP3000, simulated 3000 meters, 14.4% O_2_), and hypoxia 4000 (HYP4000, simulated 4000 meters, 12.7% O_2_) groups. During the 7-day modeling experiment, the control group was housed in a normoxic environment (21% O_2_), whereas the HYP3000 group and HYP4000 group were housed in hypoxic incubators (OX-100HE-L, TOW-INT TECH Co., Ltd., Shanghai, China) simulating oxygen concentrations at altitudes of 3000 m (14.4% O_2_) and 4000 m (12.7% O_2_), respectively [18, 19].

### 2.3 Behavioral Testing

After the modeling experiment, the open field test (OFT), tail suspension test (TST), and forced swim test (FST) were administered to the mice in order of increasing stimulus intensity to assess depressive-like behaviors [20]. For the OFT, each mouse was placed at the center of a 525 × 525 × 415 mm arena. Within 5 minutes, behaviors—including immobility time, total movement distance, center residence time, and center movement distance—were recorded [21]. In the TST, each mouse’s tail was fixed to the device with medical tape for a 6-minute session; the duration of struggling in the final 4 minutes was documented [22]. For the FST, each mouse was placed in an open-top cylindrical glass container (16 cm water depth), with the water temperature maintained at approximately 23 ± 1 °C. Following 6 minutes of swimming, the duration of struggling in the last 4 minutes was recorded [23].

### 2.4 Hematoxylin and Eosin (H&E) Staining

After the behavioral experiments were performed, the mice were anesthetized via inhalation of isoflurane (standardized dosage: 3%–5% isoflurane in medical air for induction, 1%–2% for maintenance) via a closed anesthesia chamber (R510-29, Shenzhen RWD Life Science Co., Ltd., Shenzhen, Guangdong, China). Anesthesia depth was verified by the absence of a withdrawal reflex to pinch the paw and a stable respiratory rate. Ocular enucleation was performed to collect blood samples, and the mice were subsequently euthanized by cervical dislocation (consistent with the American Veterinary Medical Association (AVMA) Guidelines for the Euthanasia of Animals). The mice were subsequently dissected to harvest tissues, and the colon and brain were used for H&E analysis. The colon and brain were fixed in 4% paraformaldehyde (HC0892, Guangzhou Mutual Success Technology Co., Ltd., Guangzhou, Guangdong, China) for 24 hours, followed by paraffin (39601095, Leica Biosystem Richmond, Inc., Richmond, IL, USA) embedding. The paraffin-embedded tissues were subsequently cut into 5 µm-thick sections and subjected to H&E staining. Pathological alterations in the tissues were observed under a light microscope and images were captured [24].

### 2.5 Enzyme-Linked Immunosorbent Assay (ELISA) for Inflammatory Factors, Oxidative Stress Indicators, and HPA Axis Indicators

Mouse blood samples were centrifuged at 4 °C and 3000 rpm for 10 minutes to obtain serum. The cerebral cortex, hypothalamus, and a portion of the colon were collected, placed in phosphate-buffered saline (PBS), and homogenized via a tissue homogenizer (Scientz-48, Ningbo Scientz Biotechnology Co., Ltd., Ningbo, Zhejiang, China) at 60 Hz for 2 minutes, after which the homogenate was centrifuged at 4 °C and 5000 rpm for 10 minutes to collect the supernatant—both the serum and the supernatant were stored at –80 °C until subsequent detection. ELISA kits (sourced from Quanzhou Jiubang Biotechnology Co., Ltd., Quanzhou, Fujian, China) were used to determine the serum levels of multiple indicators, including inflammatory factors (lipopolysaccharide (LPS, Cat# 10397), tumor necrosis factor-alpha (TNF-α, Cat# 10225), interleukin (IL)-1β (Cat# 10247), IL-6 (Cat# 10260), IL-10 (Cat# 10235), and calprotectin (CALP, Cat# 10422)), oxidative stress markers (superoxide dismutase (SOD, Cat# 10931), glutathione (GSH, Cat# 10319), and nitric oxide synthase (NOS, Cat# 15889)), vascular endothelial growth factor (VEGF, Cat# 10379), erythropoietin (EPO, Cat# 10320), and core HPA axis indicators (corticotropin-releasing hormone (CRH, Cat# 10208), corticosterone (CORT, Cat# 10352), and adrenocorticotropic hormone (ACTH, Cat# 10209)); in the colon, the inflammatory factors (LPS, TNF-α, IL-1β, IL-6, IL-10, and CALP) and hypoxia-inducible factors (HIF)-1alpha (Cat# 12247), HIF-2 (Cat# 11814), and HIF-2alpha (Cat# 11815) were assessed; and in the cerebral cortex, the neurotransmitters dopamine (DA, Cat# 15684), gamma-aminobutyric acid (GABA, Cat# 10279), BDNF (Cat# 10344), 5-hydroxytryptamine (5-HT, Cat# 10263), and norepinephrine (NE, Cat# 14758) were assessed.

### 2.6 16S Ribosomal RNA (16S rRNA) Analysis

DNA extraction from mouse colonic contents was performed with the Mag-Bind Soil DNA Kit (M5636-02, Omega Bio-tek, Inc., Norcross, GA, USA). The V3-V4 hypervariable region of the 16S rRNA gene was amplified via polymerase chain reaction (PCR). Following purification and quantification of the obtained PCR amplicons, a small-fragment library was constructed, and paired-end sequencing was performed on the Illumina NovaSeq 600 sequencing platform. The sample species composition was determined through read assembly, filtering, denoising, species annotation, and abundance analysis. The raw data (raw data) generated from sequencing contained a certain proportion of contaminated data. To ensure the accuracy and reliability of the subsequent bioinformatics analysis, quality control analyses, including quality filtering, denoising, read assembly, and chimera removal, were first performed on the raw data via default parameters in QIIME 2 2024.10 (QIIME 2 Development Team, Flagstaff, AZ, USA). Additionally, sequences with a cumulative abundance of less than 10 across all samples were filtered out, yielding amplicon sequence variants (ASVs). On the basis of rarefied ASVs, multiple diversity index analyses for ASVs and assessments of sequencing depth were conducted [25].

### 2.7 Metabolite Analysis

Untargeted metabolomics was used to identify differentially abundant metabolites in mouse fecal samples and hippocampal tissues, with chromatographic separation performed on an ultrahigh-performance liquid chromatography (UHPLC) system (Thermo Ultimate 3000, Thermo Fisher Scientific, Waltham, MA, USA) equipped with an ACQUITY UPLC® HSS T3 column (2.1 × 100 mm, 1.8 µm, Waters, Milford, MA, USA) and key parameters set as follows: flow rate = 0.3 mL/min, column temperature = 40 °C, and injection volume = 2 µL; for positive ion mode, mobile phases included 0.1% formic acid in acetonitrile (Phase B) and 0.1% formic acid in water (Phase A) with a gradient elution program of 0–1 min (10% B), 1–5 min (linear gradient to 98% B), 5–6.5 min (98% B), 6.5–6.6 min (linear gradient back to 10% B), and 6.6–8 min (10% B), while negative ion mode used acetonitrile (Phase D) and 5 mM ammonium formate in water (Phase C) with the same gradient elution program as the positive mode but replaced Phase B with Phase D [26, 27].

### 2.8 Immunofluorescence Histochemistry Assay

Paraffin-embedded mouse brains were cut into 5 µm-thick sections. After dewaxing and antigen retrieval, the sections were blocked with goat serum (1:9, AR1009, Boster Biological Technology Co., Ltd., Wuhan, Hubei, China) at room temperature for 20 minutes and then incubated overnight with primary antibodies against oligodendrocyte transcription factor 2 (Olig2) (1:1000, ab109186, Abcam, Waltham, MA, USA) and neuron–glial antigen 2 (NG2) (1:100, ab259324, Abcam). After the samples were washed, Fluorescein Isothiocyanate- (FITC; 1:300, GB22303, Servicebio, Wuhan, Hubei, China) and Cy3-conjugated (1:300, GB21301, Servicebio) secondary antibodies were added, followed by incubation at 37 °C for 30 minutes. Finally, the sections were mounted in Fluoroshield mounting medium (ab104139, Abcam) containing 4^′^,6-diamidino-2-phenylindole (DAPI). Images were acquired via a fluorescence microscope (VS200, OlyVIA, Olympus, Tokyo, Japan) [28].

### 2.9 Statistical Methods

All the data are presented as the means ± standard deviations (SDs). Differences between means were analyzed via SPSS 23.0. (IBM Corp., Armonk, NY, USA) One-way analysis of variance (one-way ANOVA), which is the appropriate statistical method for evaluating differences across multiple independent groups, was used to compare means. Levene’s test was used to assess the homogeneity of variance, and Spearman’s correlation analysis was performed. Graphs were generated via Origin software (version 2024; OriginLab Corporation, Northampton, MA, USA). Statistical significance was set at *p* < 0.05.

## 3. Results

### 3.1 Effects of Hypoxic Exposure at Different Altitudes on Mouse Behavior

**Fig. 1.**
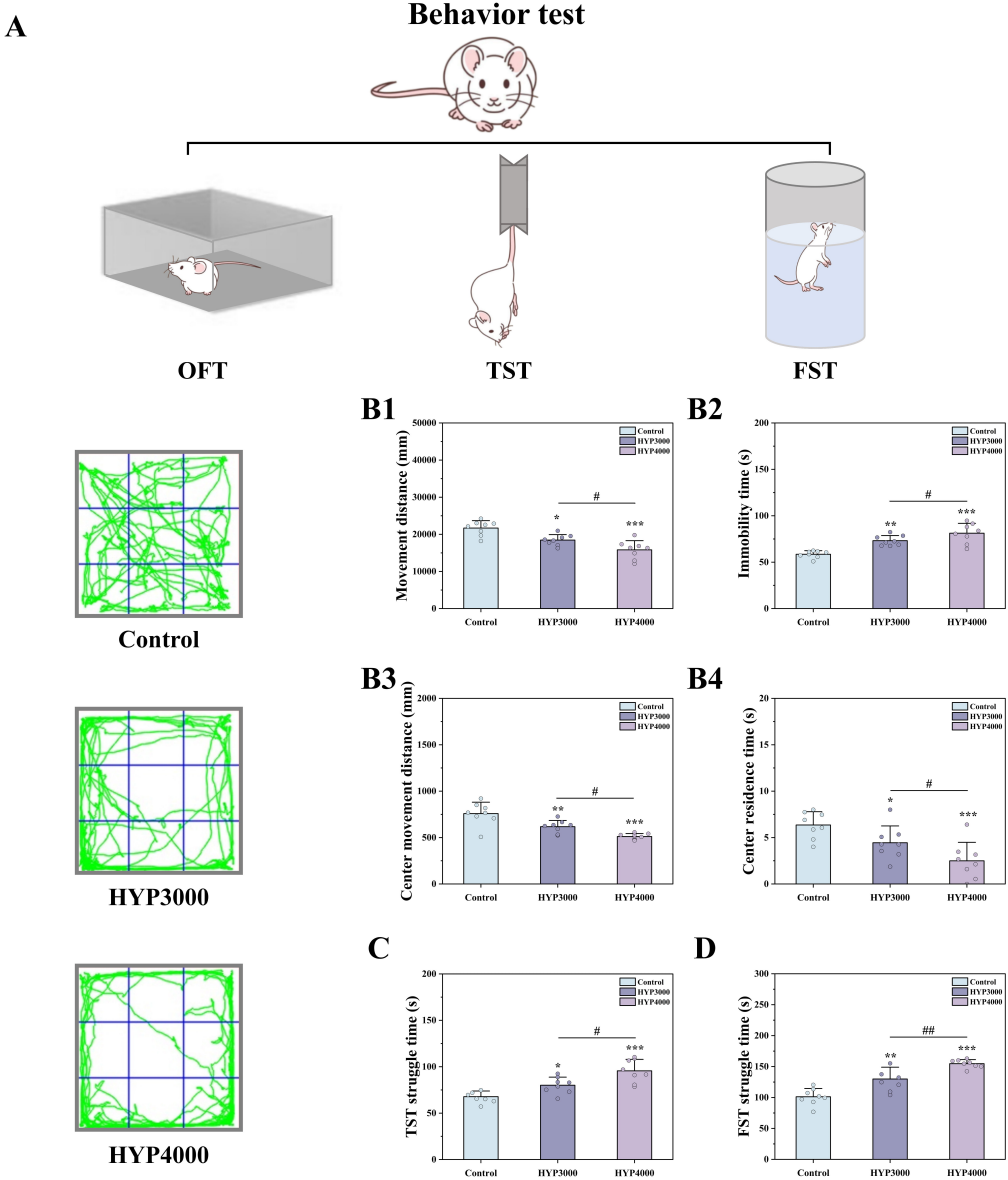
**Effects of hypoxic exposure at different altitudes on mouse behavior**. (A) Overview of the behavioral tests, (B) Open field test (OFT); (B1) Movement distance, (B2) Immobility time, (B3) Center movement distance, (B4) Center residence time, (C) Tail suspension test (TST), and (D) Forced swim test (FST). The data are presented as the means ± standard deviations (SDs) (sample size n = 8). Statistical significance is indicated as follows: *p < 0.05, **p < 0.01, and ***p < 0.001 represent differences vs. the control group; #*p* < 0.05 and ##*p* < 0.01 represent differences vs. the HYP3000 group. HYP, hypoxia.

Three behavioral tests (OFT, TST, and FST) were performed to assess depressive-like behaviors in mice exposed to 7-day simulated hypoxic environments (3000 m, 14.4% O_2_; 4000 m, 12.7% O_2_), and the results are presented in Fig. 1. Anxiety-like behaviors were assessed by the OFT. Compared with control mice, hypoxic mice presented significant reductions in central zone residence time (HYP3000: –52.31%, *p* < 0.01; HYP4000: –60.72%, *p* < 0.001) and central zone entry frequency (HYP3000: –38.46%, *p* < 0.05; HYP4000: –46.15%, *p* < 0.01), which are classic indicators of anxiety-like responses reflecting enhanced risk avoidance and reduced exploratory willingness. The total movement distance also decreased (HYP4000: –25.05%, *p* < 0.05) and was potentially associated with both motor activity suppression and anxiety-induced behavioral inhibition. Depression-like behaviors were assessed by the TST/FST. Hypoxic mice exhibited dysregulated stress coping responses, increased struggle time (TST: HYP3000: +41.23%, *p* < 0.01; FST: HYP3000: +37.58%, *p* < 0.01) and decreased immobility time (TST: HYP4000: –28.36%, *p* < 0.001; FST: HYP4000: –23.71%, *p* < 0.01). This paradoxical phenotype, which is distinct from conventional antidepressant-like responses, reflects impaired stress adaptation rather than emotional improvement, as contextualized by concurrent neurobiological damage.

### 3.2 Effects of Hypoxic Exposure at Different Altitudes on Mouse Hippocampal, Colon, and Adipose Tissues

**Fig. 2.**
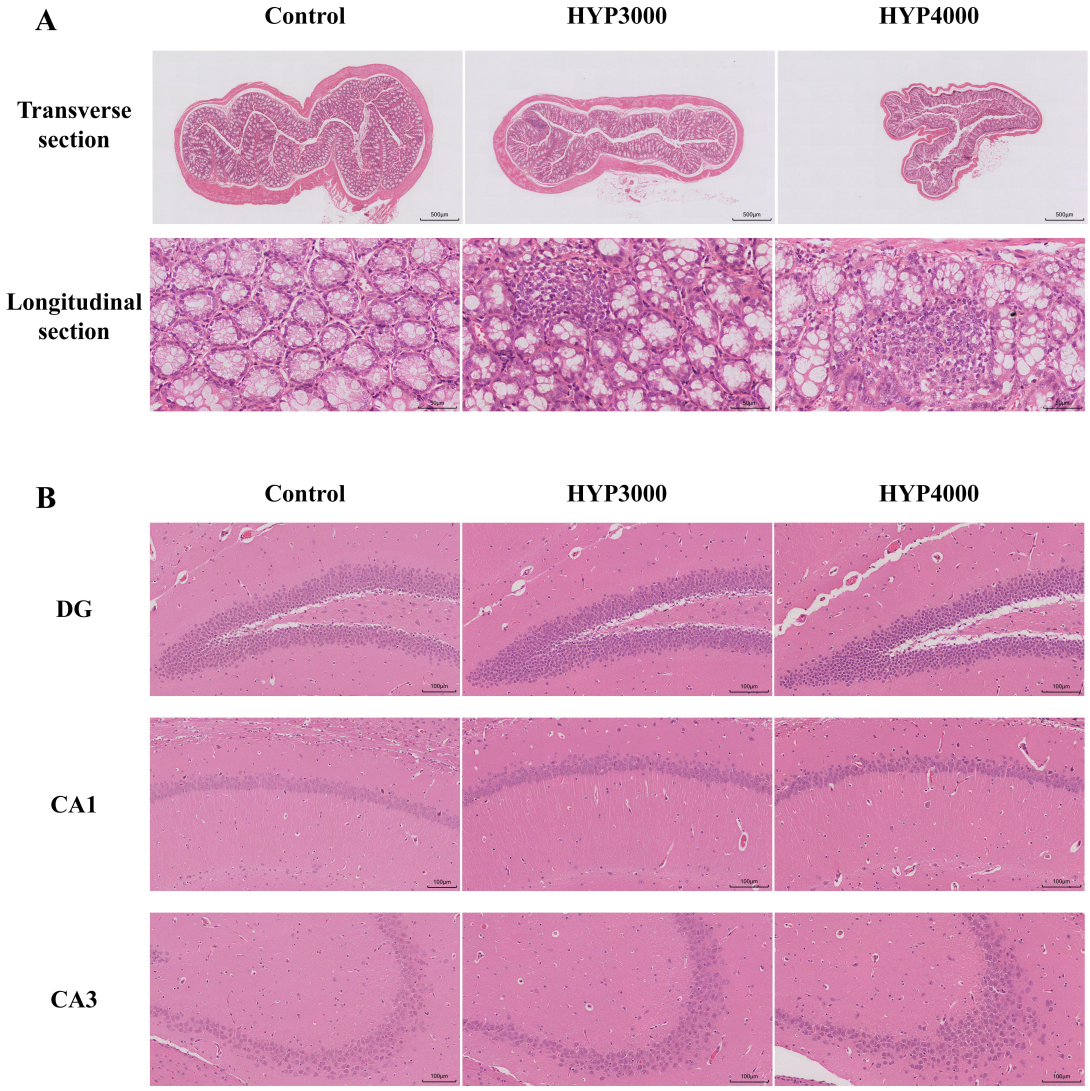
**Effects of hypoxic exposure at different altitudes on the hippocampus and colon tissue of mice**. (A) Transverse (scale bar = 500 µm) and longitudinal (scale bar = 50 µm) sections of colon tissue. (B) 100 µm sections of cells in the hippocampal dentate gyrus (DG), Cornu Ammonis 1 (CA1), and Cornu Ammonis 3 (CA3) subregions; scale bar = 100 µm.

H&E staining was performed on the colon and hippocampal tissues of the mice, as shown in Fig. 2. In the control group, the colonic tissue structure showed clear and complete layers; the epithelial cells had a regular morphology and were closely arranged, with no obvious inflammatory cell infiltration observed. In contrast, all the hypoxic groups exhibited varying degrees of inflammatory damage; the epithelial cells in the mucosal layer were disorganized, some cells showed degeneration or even necrosis, and obvious inflammatory cell infiltration was observed.

In the control group, the mice exhibited intact hippocampal subregion structures: the neuronal nuclei had clear boundaries, and the cells were arranged in a distinct layered pattern. In contrast, in the hypoxic groups, the cells in the hippocampal dentate gyrus (DG) region showed intense staining (with some cells swollen or deformed); no obvious pathological changes were observed in the Cornu Ammonis 1 (CA1) region, but cells in the Cornu Ammonis 3 (CA3) region were disorganized, loosely arranged, and had wider intercellular spaces.

### 3.3 Effects of Hypoxic Exposure at Different Altitudes on Inflammation in Mice

**Fig. 3.**
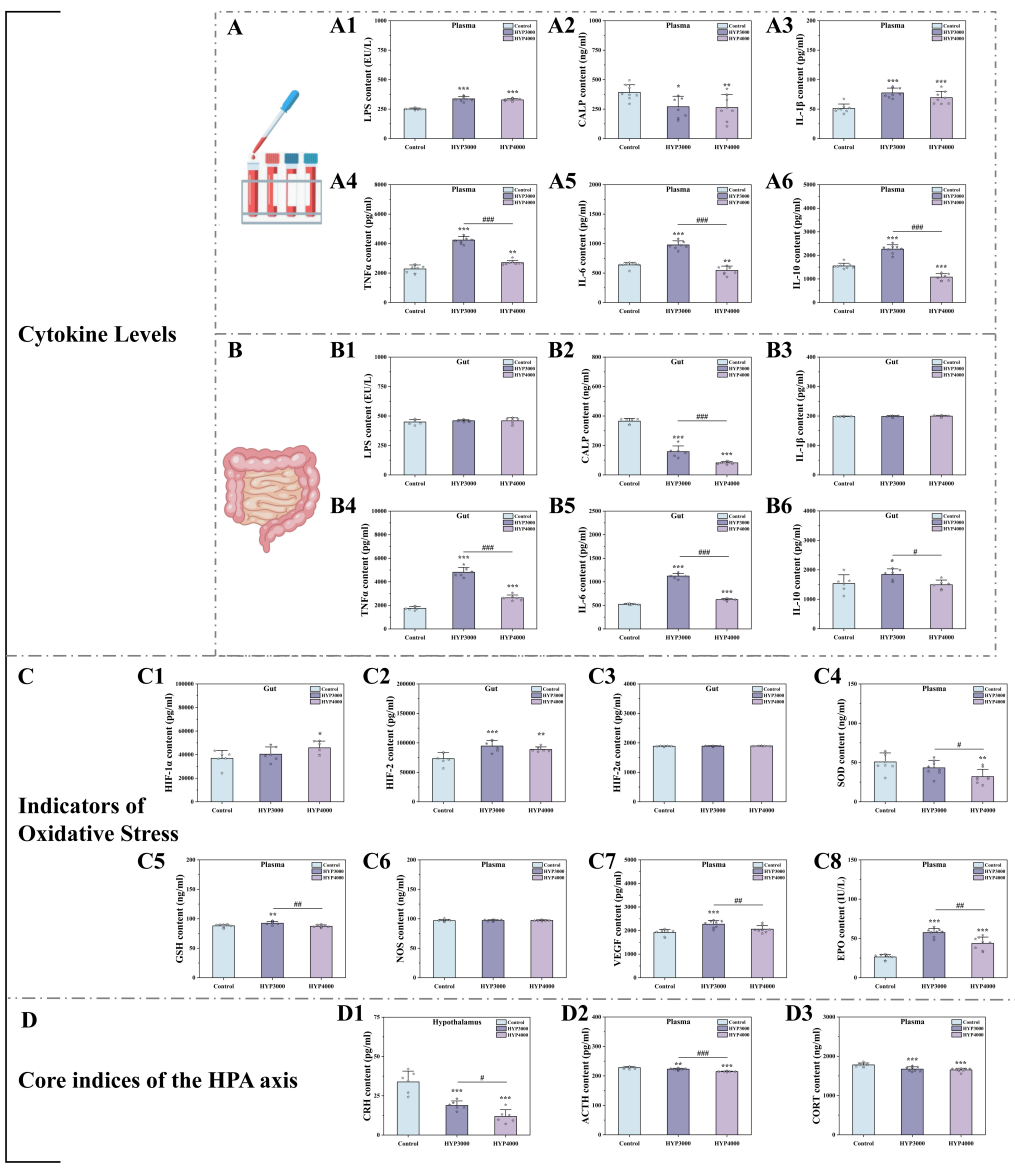
**Effects of hypoxic exposure at different altitudes on inflammatory markers, oxidative stress, and the hypothalamic‒pituitary‒adrenal (HPA) axis**. (A) Serum inflammatory indicators, (A1–A6) are as follows: Lipopolysaccharide (LPS); calprotectin (CALP); interleukin (IL) -1β; tumor necrosis factor-alpha (TNF-α), IL-6, IL-10. (B) colonic inflammatory indicators, (B1–B6) are as follows: LPS, CALP, IL-1β, TNF-α, IL-6, IL-10. (C) oxidative stress indicators, (C1–C8) are as follows: hypoxia-inducible factor (HIF)-1alpha, HIF-2, HIF-2alpha, superoxide dismutase (SOD), glutathione (GSH), nitric oxide synthase (NOS), vascular endothelial growth factor (VEGF), erythropoietin (EPO). (D) HPA axis indicators, (D1–D3) are as follows: corticotropin-releasing hormone (CRH), adrenocorticotropic hormone (ACTH), corticosterone (CORT). The data are presented as the means ± SDs, with sample sizes of n = 8 (plasma) and n = 6 (gut). Statistical significance is denoted as follows: *p < 0.05, **p < 0.01, and ***p < 0.001 indicate differences vs. the control group; #*p* < 0.05, ##*p* < 0.01, and ###*p* < 0.001 indicate differences vs. the HYP3000 group.

We detected inflammatory indicators in mouse serum and colonic tissues (results in Fig. 3A,B). Compared with those in the control group, the serum levels of LPS (HYP3000: +34.20%, *p* < 0.001; HYP4000: +30.86%, *p* < 0.001), TNF-α (HYP3000: +86.14%, *p* < 0.001; HYP4000: +18.84%, *p* < 0.01) and IL-1β (HYP3000: +51.70%, *p* < 0.001; HYP4000: +25.81%, *p* < 0.001) were significantly elevated, whereas the levels of IL-6 (HYP3000: –53.24%, *p* < 0.001; HYP4000: –14.46%, *p* < 0.01), IL-10 (HYP3000: –46.12%, *p* < 0.001; HYP4000: –30.42%, *p* < 0.001) and CALP (HYP3000: –31.01%, *p* < 0.05; HYP4000: –32.73%, *p* < 0.01) were notably lower, compared with HYP3000, HYP4000 showed no significant changes in serum LPS (–2.49%), IL-1β (–10.48%) or CALP (–2.41%), but significantly lower levels of TNF-α (–36.15%, *p* < 0.001), IL-6 (–44.18%, *p* < 0.001) and IL-10 (–22.38%, *p* < 0.001). For colonic tissues, compared with the control group, LPS was increased (HYP3000: +2.29%; HYP4000: +2.29%), TNF-α (HYP3000: +174.96%, *p* < 0.001; HYP4000: +51.02%, *p* < 0.001) and IL-1β (HYP3000: +0.01%; HYP4000: +0.59%) were elevated, IL-6 (HYP3000: +114.30%, *p* < 0.001; HYP4000: +18.90%, *p* < 0.001) and IL-10 (HYP3000: +20.22%, *p* < 0.05; HYP4000: +2.72%) were significantly increased, and CALP (HYP3000: –56.70%, *p* < 0.001; HYP4000: –77.58%, *p* < 0.001) was markedly reduced. Compared with HYP3000, HYP4000 was not significantly different in colonic LPS or IL-1β, but significantly lower levels of TNF-α (–45.08%, *p* < 0.001), IL-6 (–44.52%, *p* < 0.001), IL-10 (–19.08%, *p* < 0.05) and CALP (–48.22%, *p* < 0.001) were observed.

### 3.4 Effects of Hypoxic Exposure at Different Altitudes on Oxidative Stress Levels in Mice

Oxidative stress indicators (SOD, GSH, NOS, VEGF, and EPO) in mouse serum and hypoxia-inducible factors (HIF-1alpha, HIF-2, and HIF-2alpha) in the colon were detected, and the results are shown in Fig. 3C. Compared with those in the control group, the levels of SOD (HYP3000: –14.89%; HYP4000: –36.79%, *p* < 0.01) were lower; the levels of GSH (HYP3000: 5.13%, *p* < 0.01; HYP4000: –0.70%), NOS (HYP3000: 0.20%; HYP4000: –0.44%), VEGF (HYP3000: 18.29%, *p* < 0.001; HYP4000: 7.23%) and EPO (HYP3000: 117.87%, *p* < 0.001; HYP4000: 65.88%, *p* < 0.001) were greater; the levels of HIF-1alpha (HYP3000: 9.61%; HYP4000: 24.21%, *p* < 0.05) and HIF-2 (HYP3000: 29.25%, *p* < 0.001; HYP4000: 21.53%, *p* < 0.01) were significantly greater; and the levels of HIF-2alpha (HYP3000: 0.18%; HYP4000: 0.46%) were greater.

Compared with those in the HYP3000 group, the levels of SOD (–25.74%, *p* < 0.05), GSH (–5.54%, *p* < 0.01), VEGF (–9.35%, *p* < 0.01) and EPO (–23.86%, *p* < 0.001) were significantly lower; the levels of HIF-1alpha (13.31%) and HIF-2alpha (0.27%) were greater; and the levels of HIF-2 (–5.97%) were lower in the HYP4000 group.

### 3.5 Effects of Hypoxic Exposure at Different Altitudes on the HPA Axis in Mice

We assessed core HPA axis indicators (CRH, CORT, and ACTH) and the results are shown in Fig. 3D. Compared with the control group, the hypoxia groups (HYP3000 and HYP4000) presented significantly lower levels of CORT (HYP3000: –5.95%, *p* < 0.001; HYP4000: –7.12%, *p* < 0.001), ACTH (HYP3000: –2.12%, *p* < 0.05; HYP4000: –5.83%, *p* < 0.001), and CRH (HYP3000: –44.28%, *p* < 0.001; HYP4000: –64.92%, *p* < 0.001). Compared with the HYP3000 group, the HYP4000 group presented lower levels of CORT (–1.25%), ACTH (–3.79%, *p* < 0.001), and CRH (–37.04%, *p* < 0.05).

### 3.6 Effects of Hypoxic Exposure at Different Altitudes on Mouse Gut Microbiota Diversity

#### 3.6.1 Alpha and Beta Diversity Analyses

**Fig. 4.**
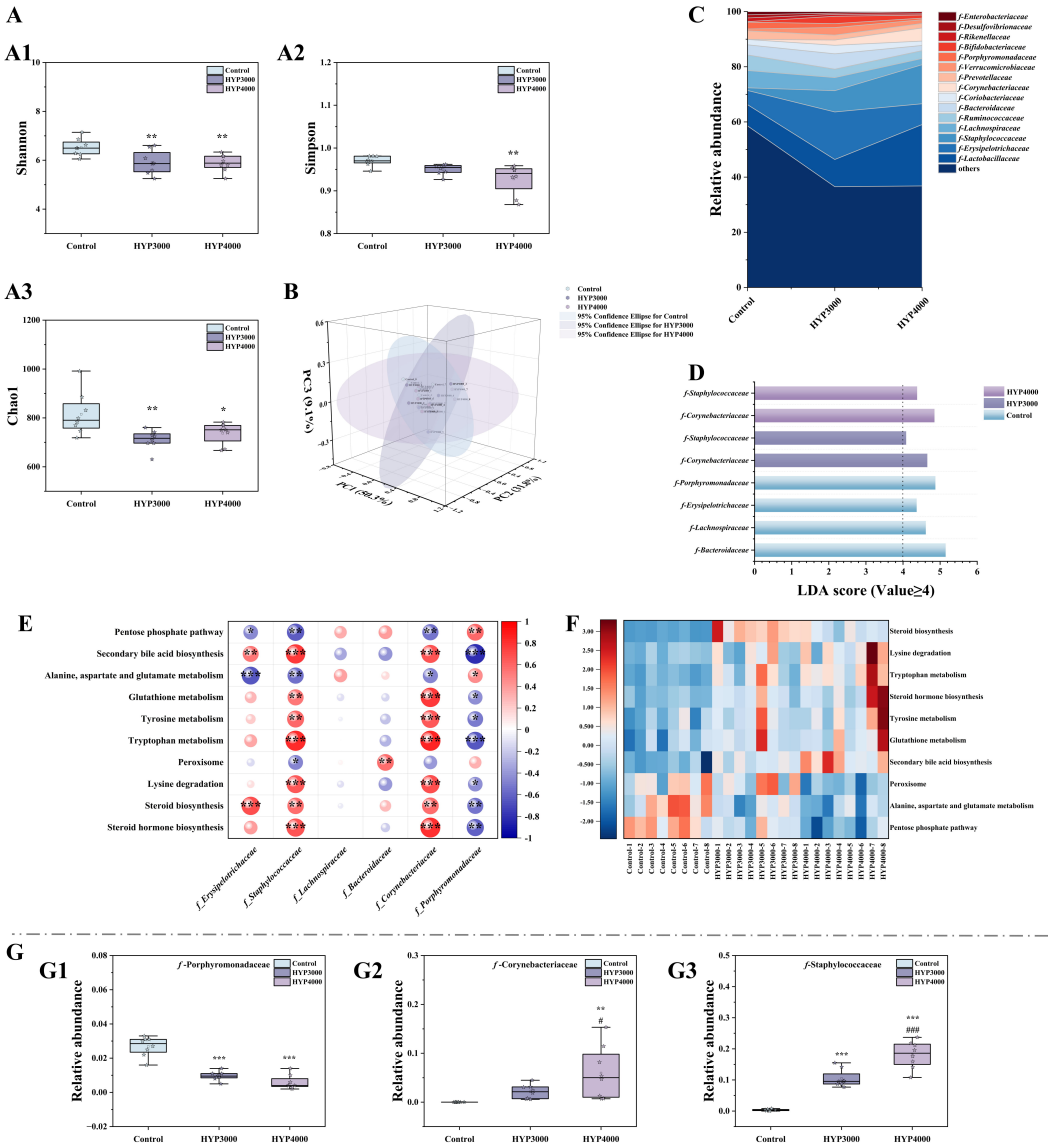
**Effects of hypoxic exposure at different altitudes on gut microbiota diversity in mice**. (A) α diversity analysis, (A1–A3) are as follows: Shannon, Simpson, and Chao1. (B) β diversity analysis, (C) analysis of the top 10 Kyoto Encyclopedia of Genes and Genomes (KEGG) pathways, (D) correlation analysis between functional pathways and differentially abundant gut microbiota taxa, (E) KEGG functional prediction (level 3), and (F) family-level compositional analysis of the gut microbiota. (G) Relative abundance of key microbiota, (G1–G3) are as follows: *f-Porphyromonadaceae, f-Corynebacteriaceae, f-Staphylococcaceae*. The data are presented as the means ± SDs (n = 8). Statistical significance is indicated as follows: *p < 0.05, **p < 0.01, and ***p < 0.001 represent differences vs. the control group; #*p* < 0.05 and ###*p* < 0.001 represent differences vs. the HYP3000 group.

Analysis of the alpha diversity of the gut microbiota (Fig. 4A) revealed that compared with the control group, the hypoxia group presented significantly lower Shannon (HYP3000: *p* < 0.01; HYP4000: *p* < 0.05), Simpson (HYP4000: *p* < 0.05), and Chao1 (HYP3000: *p* < 0.01; HYP4000: *p* < 0.01) indices. No significant differences in alpha diversity indices were detected between the HYP3000 and HYP4000 groups. With respect to beta diversity, principal coordinate analysis (PCoA) based on Bray‒Curtis distances (Fig. 4B) revealed differences in the composition and structure of the gut microbiota between the control group and the hypoxia groups (PC1: 50.3%, PC2: 31.8%, PC3: 9.1%).

#### 3.6.2 Linear Discriminant Analysis Effect Size (LEfSe) Analysis

Analysis of relative abundance at the family level (Fig. 4C) revealed that among the top 15 most abundant taxa, *f-Lactobacillaceae* (Control: 7.71%; HYP3000: 9.87%; HYP4000: 22.25%), *f-Erysipelotrichaceae* (Control: 5.14%; HYP3000: 17.23%; HYP4000: 7.54%), *f-Staphylococcaceae* (Control: 0.97%; HYP3000: 7.70%; HYP4000: 14.16%), *f-Corynebacteriaceae* (Control: 0.01%; HYP3000: 1.89%; HYP4000: 4.73%), *f-Verrucomicrobiaceae* (Control: 0.67%; HYP3000: 2.74%; HYP4000: 1.36%), and *f-Bifidobacteriaceae* (Control: 0.19%; HYP3000: 2.66%; HYP4000: 1.12%) were increased in the hypoxic groups. In contrast, the abundances of *f-Lachnospiraceae* (Control: 6.27%; HYP3000: 4.74%; HYP4000: 2.25%), *f-Ruminococcaceae* (Control: 5.64%; HYP3000: 3.06%; HYP4000: 2.86%), *f-Prevotellaceae* (Control: 3.12%; HYP3000: 1.80%; HYP4000: 1.63%), *f-Porphyromonadaceae* (Control: 2.48%; HYP3000: 1.41%; HYP4000: 0.60%), *f-Rikenellaceae* (Control: 1.35%; HYP3000: 0.68%; HYP4000: 0.43%), *f-Desulfovibrionaceae* (Control: 1.14%; HYP3000: 0.50%; HYP4000: 0.79%), and *f-Enterobacteriaceae* (Control: 1.14%; HYP3000: 0.52%; HYP4000: 0.00%) were decreased in the hypoxic groups.

Subsequent LEfSe analysis revealed taxa contributing significantly to the intergroup differences, with a linear discriminant analysis (LDA) score threshold of 4 (Fig. 4D). There were 4, 2, and 2 statistically distinct taxa in the control, HYP3000, and HYP4000 groups, respectively, all of which were among the 15 most abundant taxa.

#### 3.6.3 Kyoto Encyclopedia of Genes and Genomes (KEGG) Functional Prediction of the Microbiota

KEGG functional prediction at level 3 (Fig. 4E) revealed the top 10 differential pathways, which included the pentose phosphate pathway, secondary bile acid biosynthesis, alanine-aspartate-glutamate metabolism, glutathione metabolism, tyrosine metabolism, tryptophan metabolism, peroxisome, lysine degradation, steroid biosynthesis, and steroid hormone biosynthesis, and Spearman correlation analysis (Fig. 4F), between LEfSe-identified differentially abundant taxa, and these pathways revealed that *f-Staphylococcaceae* and *f-Corynebacteriaceae* were significantly positively correlated with secondary bile acid biosynthesis (both *p* < 0.001), glutathione metabolism (both *p* < 0.01), tyrosine metabolism (*p* < 0.01 and *p* < 0.001, respectively), tryptophan metabolism (both *p* < 0.001), lysine degradation (both *p* < 0.001), steroid biosynthesis (both *p* < 0.01), and steroid hormone biosynthesis (both *p* < 0.001) but were significantly negatively correlated with the pentose phosphate pathway (both *p* < 0.01) and alanine-aspartate-glutamate metabolism (both *p* < 0.01), whereas *f-Porphyromonadaceae* was significantly negatively correlated with secondary bile acid biosynthesis (*p* < 0.001), glutathione metabolism (*p* < 0.05), and tyrosine metabolism (*p* < 0.05).

#### 3.6.4 Analysis of Differential Relative Abundance at the Family Level

Further analysis of *f-Staphylococcaceae*, *f-Corynebacteriaceae*, and *f-Porphyromonadaceae* (Fig. 4G) revealed that, compared with the control, the hypoxic groups presented significantly more *f-Staphylococcaceae* (HYP3000: *p* < 0.001; HYP4000: *p* < 0.001) and *f-Corynebacteriaceae* (HYP3000: *p* = 0.20; HYP4000: *p* < 0.01) and significantly fewer *f-Porphyromonadaceae* (HYP3000: *p* < 0.001; HYP4000: *p* < 0.001). Compared with the HYP3000 group, the HYP4000 group presented significantly more *f-Staphylococcaceae* (*p* < 0.001) and *f-Corynebacteriaceae* (*p* < 0.05).

### 3.7 Effects of Hypoxic Exposure at Different Altitudes on Gut Metabolism in Mice

#### 3.7.1 Principal Component Analysis (PCA) and Differentially Abundant Metabolite (DM) Analysis

**Fig. 5.**
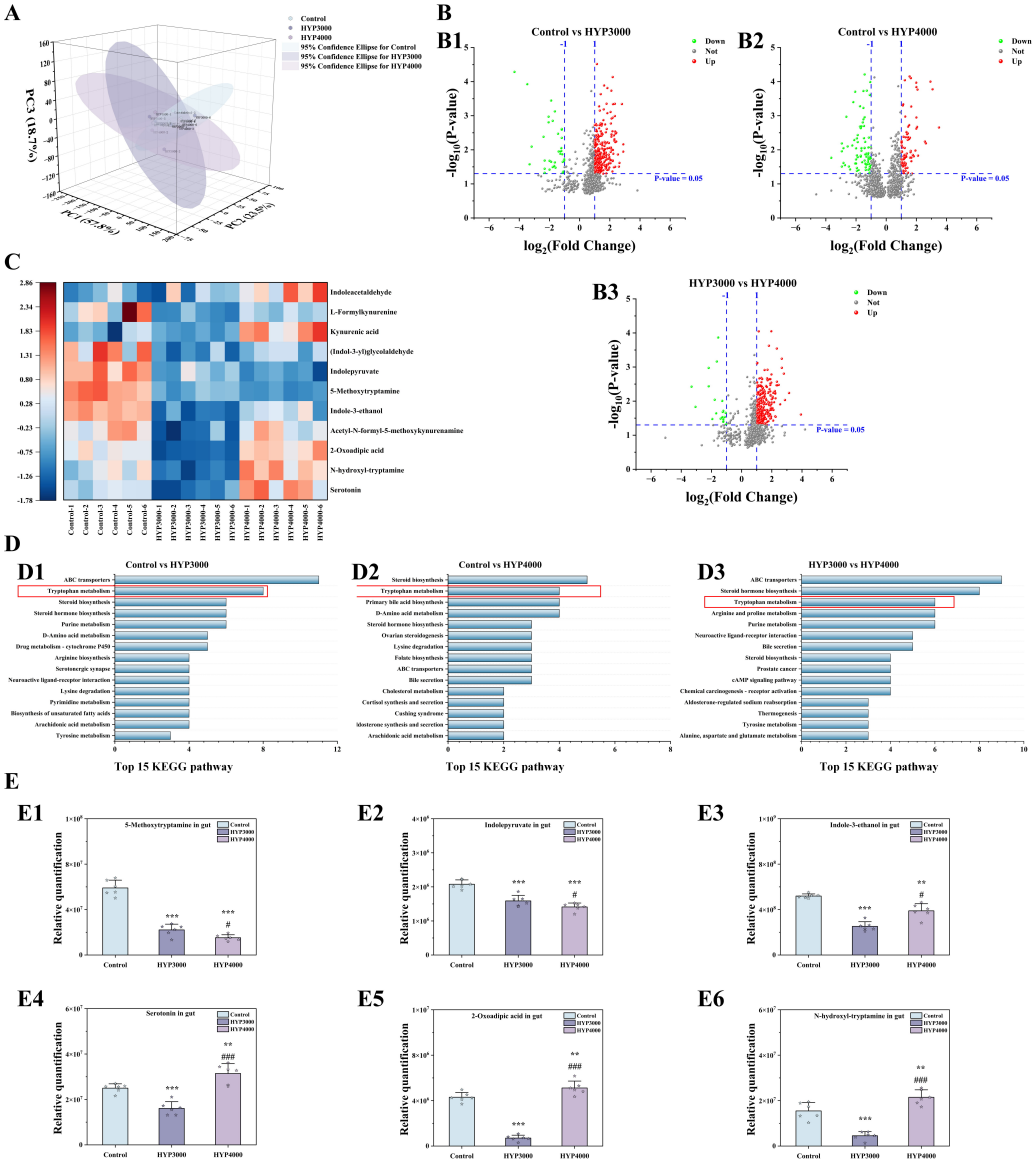
**Effects of hypoxic exposure at different altitudes on gut metabolism in mice**. (A) Principal component analysis (PCA). (B) volcano plots, (B1–B3) are as follows: comparison between Control and the HYP3000, comparison between Control and the HYP4000, comparison between HYP3000 and the HYP4000. (C) analysis of differentially abundant metabolites. (D) KEGG pathway analysis, (D1–D3) are as follows: comparison between Control and the HYP3000, comparison between Control and the HYP4000, comparison between HYP3000 and the HYP4000. The red box is used to highlight the tryptophan metabolism. (E) differential metabolite abundance analysis, (E1–E6) are as follows: 5-methoxytryptamine, Indolepyruvate, Indole-3-ethanol, serotonin, 2-oxoadipic acid and N-hydroxyl-tryptamine in gut. The data are presented as the means ± SDs (sample size n = 6). Statistical significance is denoted as follows: **p < 0.01 and ***p < 0.001 indicate differences vs. the control group; #*p* < 0.05 and ###*p* < 0.001 indicate differences vs. the HYP3000 group.

Analysis of the metabolite composition in the mouse colon by principal component analysis (PCA; Fig. 5A) revealed differences in the composition and structure of the gut metabolites between the control group and hypoxic groups (PC1: 57.8%, PC2: 23.5%, and PC3: 18.7%). The volcano plot (Fig. 5B) displays differentially abundant metabolite expression between groups, with screening criteria defined as follows: VIP >1, *p* < 0.05, log_2_(fold change) <–1 (downregulation) and log_2_(fold change) >1 (upregulation). Compared with the control group, the HYP3000 group had 34 downregulated DMs and 262 upregulated DMs; the HYP4000 group had 82 downregulated DMs and 77 upregulated DMs. Compared with the HYP3000 group, the HYP4000 group presented 19 downregulated DMs and 270 upregulated DMs.

#### 3.7.2 Analyses of Differentially Enriched Pathways and Differentially Abundant Metabolites

KEGG pathway enrichment analysis (Fig. 5D) revealed tryptophan metabolism, steroid biosynthesis, and steroid hormone biosynthesis as shared pathways across all group comparisons. Among these pathways, tryptophan metabolism was among the top three pathways in the comparisons among the three groups.

Cluster analysis of DMs in this pathway revealed that, compared with those in the control group (Fig. 5C), all 11 DMs in the HYP3000 group were downregulated; in the HYP4000 group, 6 DMs were downregulated (5-methoxytryptamine, acetyl-N-formyl-5-methoxykynurenamine, L-formylkynurenine, Indolepyruvate, Indole-3-ethanol, and Indole-3-glycolaldehyde), and 5 DMs were upregulated (serotonin, N-hydroxyl-tryptamine, kynurenic acid, 2-oxoadipic acid, and Indoleacetaldehyde). Compared with the HYP3000 group, the HYP4000 group had 2 downregulated DMs (5-methoxytryptamine and Indolepyruvate) and 9 upregulated DMs (serotonin, N-hydroxyl-tryptamine, acetyl-N-formyl-5-methoxykynurenineamine, kynurenic acid, 2-oxoadipic acid, L-formylkynurenine, Indole-3-ethanol, Indole-3-glycolaldehyde, and Indoleacetaldehyde).

A detailed comparison of 6 DMs with significant differences across all three groups (Fig. 5E) revealed that, compared with the control group, the HYP3000 group presented significant decreases in 5-methoxytryptamine (–62.56%, *p* < 0.001), Indolepyruvate (–23.53%, *p* < 0.001), Indole-3-ethanol (–51.39%, *p* < 0.001), serotonin (–35.66%, *p* < 0.001), N-hydroxyl-tryptamine (–70.75%, *p* < 0.001), and 2-oxoadipic acid (–83.69%, *p* < 0.001); moreover, the HYP4000 group presented significant decreases in 5-methoxytryptamine (–74.21%, *p* < 0.001), Indolepyruvate (–32.10%, *p* < 0.001), and Indole-3-ethanol (–25.02%, *p* < 0.001), as well as significant increases in serotonin (25.86%, *p* < 0.01), N-hydroxyl-tryptamine (38.84%, *p* < 0.01), and 2-oxoadipic acid (18.98%, *p* < 0.01).

### 3.8 Effects of Hypoxic Exposure at Different Altitudes on Hippocampal Metabolism in Mice

#### 3.8.1 PCA and DMs Analyses

**Fig. 6.**
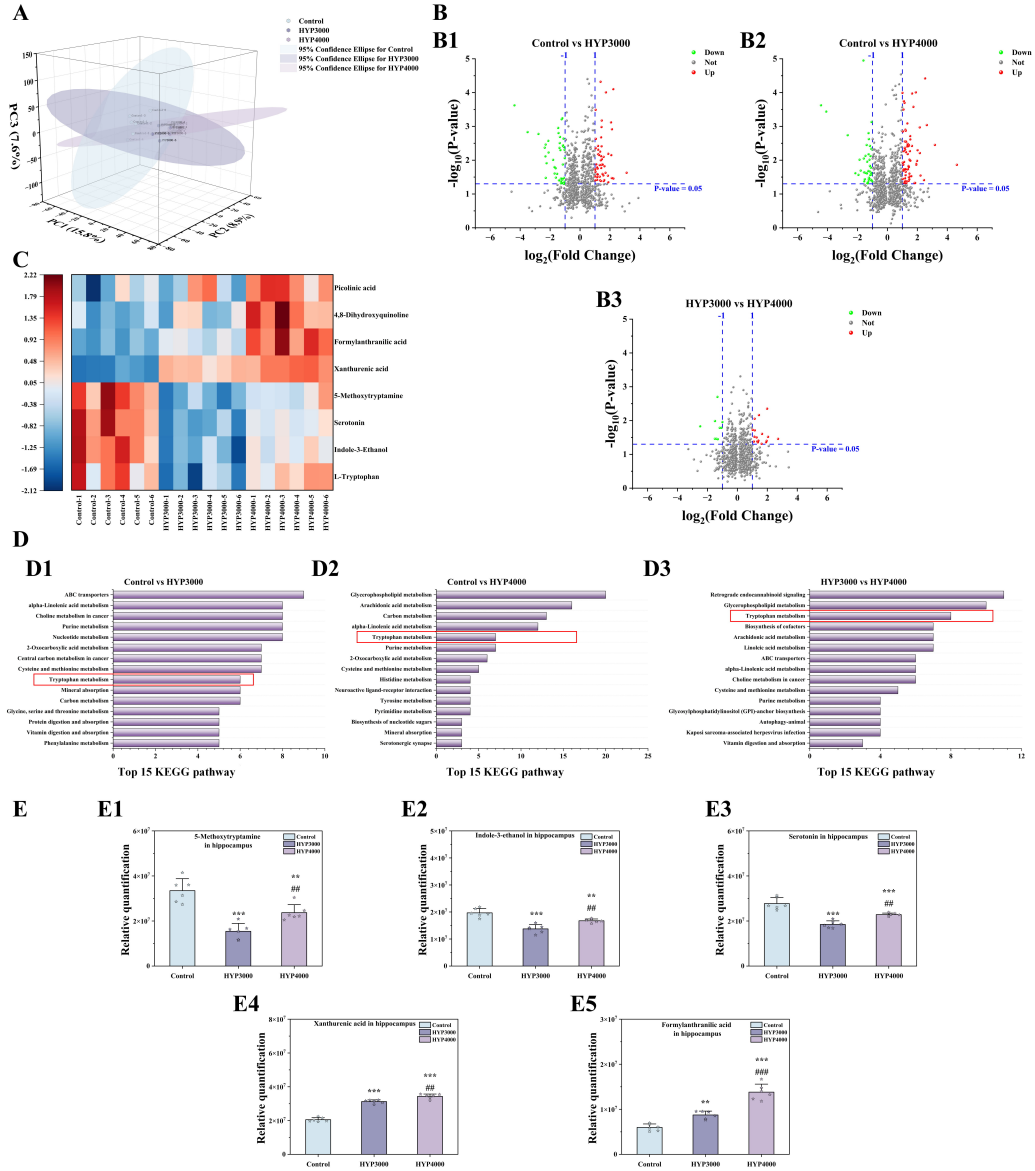
**Effects of hypoxic exposure at different altitudes on hippocampal metabolism in mice**. (A) PCA. (B) volcano plots, (B1–B3) are as follows: comparison between Control and the HYP3000, comparison between Control and the HYP4000, comparison between HYP3000 and the HYP4000. (C) analysis of differentially abundant metabolites, (D) KEGG pathway analysis, (D1–D3) are as follows: comparison between Control and the HYP3000, comparison between Control and the HYP4000, comparison between HYP3000 and the HYP4000. The red box is used to highlight the tryptophan metabolism. (E) differential metabolite abundance analysis, (E1–E5) are as follows: 5-methoxytryptamine, Indole-3-ethanol, serotonin, xanthurenic acid and formylanthranilic acid in hippocampal. The data are presented as the means ± SDs (sample size n = 6). Statistical significance is indicated as follows: **p < 0.01, ***p < 0.001 represent differences vs. the control group; ##*p* < 0.01, ###*p* < 0.001 represent differences vs. the HYP3000 group.

Analysis of untargeted metabolomics data from mouse hippocampi by PCA (Fig. 6A) revealed differences in the composition and structure of hippocampal metabolites between the control group and hypoxic groups (PC1: 15.8%, PC2: 8.9%, and PC3: 7.6%). The volcano plot of hippocampal metabolites (screening criteria: VIP >1; *p* value < 0.05; log_2_(fold change) <–1 for downregulation, log_2_(fold change) >1 for upregulation) revealed the following: compared with the control group, the HYP3000 group had 39 downregulated DMs and 51 upregulated DMs, and the HYP4000 group had 41 downregulated DMs and 69 upregulated DMs. Compared with the HYP3000 group, the HYP4000 group had 9 downregulated DMs and 19 upregulated DMs (Fig. 6B).

#### 3.8.2 Analyses of Differentially Enriched Pathways and Differentially Abundant Metabolites

KEGG pathway enrichment analysis (Fig. 6D) revealed that the tryptophan metabolism pathway was a shared pathway across all groups, which was consistent with the results of the gut metabolism analysis. Cluster analysis of the DMs in this pathway (Fig. 6C) revealed that compared with the control group, the HYP3000 group had 4 downregulated DMs (L-tryptophan, Indole-3-ethanol, serotonin, and 5-methoxytryptamine) and 4 upregulated DMs (xanthurenic acid, formylanthranilic acid, 4,8-dihydroxyquinoline, and picolinic acid); the HYP4000 group also had 4 downregulated DMs (L-tryptophan, Indole-3-ethanol, serotonin, and 5-methoxytryptamine) and 4 upregulated DMs (xanthurenic acid, formylanthranilic acid, 4,8-dihydroxyquinoline, and picolinic acid). Compared with those in the HYP3000 group, all the DMs in the HYP4000 group were upregulated.

A detailed comparison of 5 DMs with significant differences across all three groups (Fig. 6E) revealed that, compared with the control group, the HYP3000 group presented significant decreases in 5-methoxytryptamine (–53.91%, *p* < 0.001), Indole-3-ethanol (–30.11%, *p* < 0.001), and serotonin (–33.41%, *p* < 0.001), along with significant increases in xanthurenic acid (52.40%, *p* < 0.001) and formylanthranilic acid (46.19%, *p* < 0.01). Compared with the control group, the HYP4000 group presented significant decreases in 5-methoxytryptamine (–29.15%, *p* < 0.01), Indole-3-ethanol (–14.90%, *p* < 0.01), and serotonin (–17.68%, *p* < 0.001), as well as significant increases in xanthurenic acid (67.34%, *p* < 0.001) and formylanthranilic acid (130.75%, *p* < 0.001).

### 3.9 RDA and Spearman Correlation Analysis

#### 3.9.1 Redundancy Analysis/Canonical Correspondence (RDA/CCA) Analysis

RDA/CCA was performed to reduce the dimensionality of the behavioral phenotypes, biochemical indicators, gut microbiota, and DMs. The length of the arrow is positively correlated with the intensity of the factor’s influence on metabolites, whereas the straight-line distance from a metabolite to an arrow is negatively correlated with their association.

**Fig. 7.**
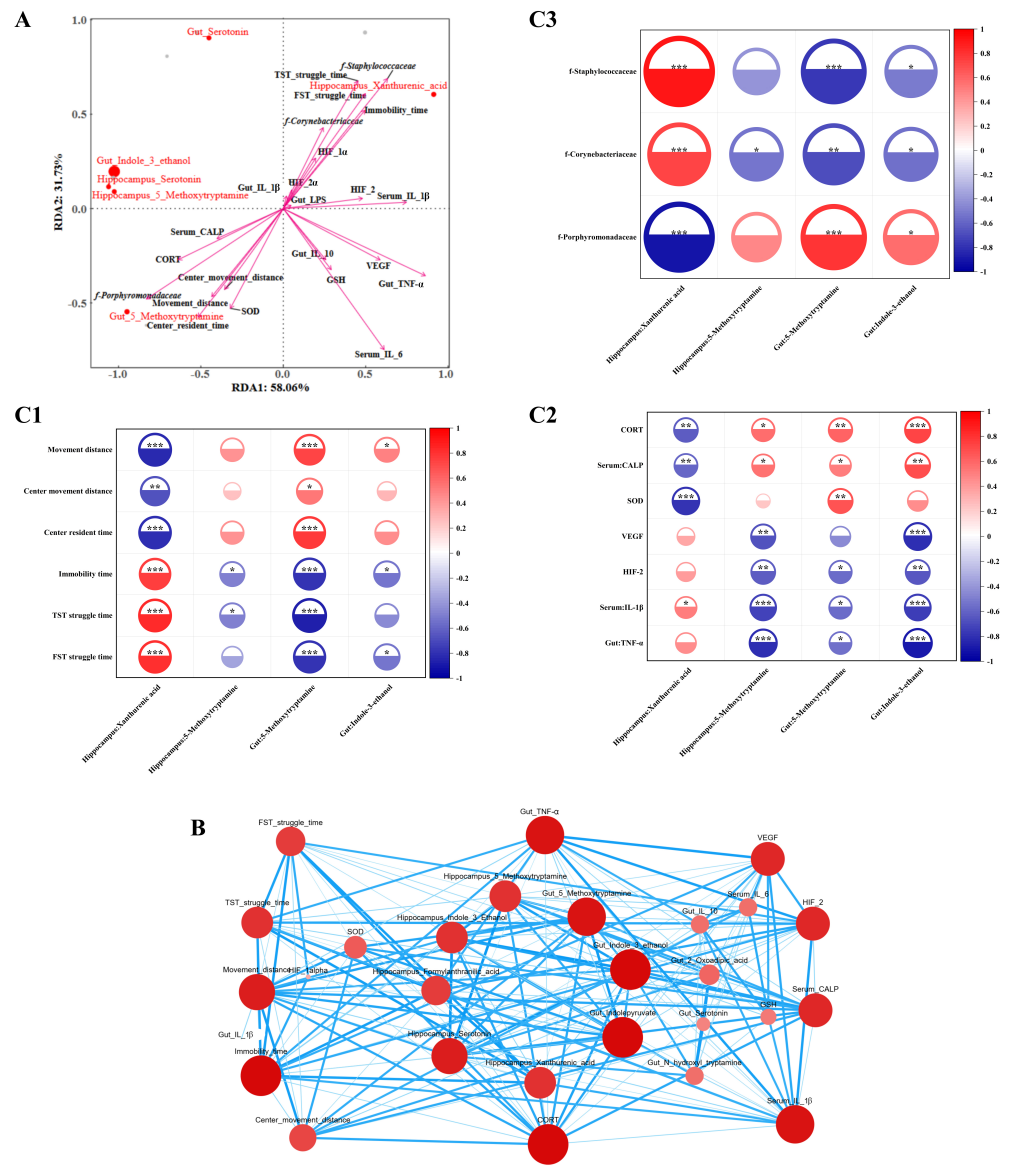
**Spearman correlation analysis and network visualization (sample size n = 6)**. (A) Redundancy analysis (RDA), (B) correlation network diagram, and (C) Spearman correlation analysis, (C1–C3) are as follows: association between metabolites and behavioral indicators, association between metabolites and biochemical indicators, association between metabolites and microbiota. In Panel B, the node size reflects the core degree of each substance (larger nodes correspond to higher core degrees), whereas the thickness of lines between nodes represents the absolute value of the correlation coefficient (thicker lines indicate stronger correlations). *p ≤0.05, **p ≤0.01, ***p ≤ 0.001.

The results (Fig. 7A) revealed that the analysis was strongly representative (explanatory rate: 89.79%; variance inflation factor (VIF) >10). The influence intensity of the differentially abundant metabolites was in the following order: Gut: Indole-3-ethanol > Gut: 5-Methoxytryptamine > Hippocampus: xanthurenic acid > Hippocampus: 5-Methoxytryptamine > Gut: Serotonin > Hippocampus: Serotonin.

The strength of the relationships between differentially abundant metabolites and behavioral phenotypes decreased in the following order: TST struggle time > FST struggle time > Center residence time > Immobility time > Movement distance > Center movement distance.

The strength of the relationships between differentially abundant metabolites and biochemical indicators was in the following order: IL-6 > Gut: TNF-α > Serum: IL-1β > CORT > VEGF > SOD > HIF-2 > GSH > Serum: CALP > Gut: IL-10 > HIF-1alpha.

The strength of the relationships between the differentially abundant metabolites and microbiota decreased in the following order: *f-Porphyromonadaceae* >*f-Staphylococcaceae* >*f-Corynebacteriaceae*.

#### 3.9.2 Network Diagram and Spearman Correlation Analysis

Network correlation analysis was conducted on behavioral phenotypes, biochemical indicators, the gut microbiota, and differentially abundant metabolites, with the results revealing four metabolites with high degree, closeness centrality, and betweenness centrality—gut: Indole-3-ethanol (degree: 19; closeness centrality: 23; betweenness centrality: 47), gut:5-methoxytryptamine (degree: 18; closeness centrality: 23; betweenness centrality: 9), hippocampus:5-methoxytryptamine (degree: 15; closeness centrality: 21; betweenness centrality: 13), and hippocampus: xanthural-acid (degree: 15; closeness centrality: 21; betweenness centrality: 5)—indicating that they are key substances connecting various indicators (Fig. 7B). Furthermore, Spearman correlation analysis between these four metabolites and key behavioral phenotypes, biochemical indicators, and the gut microbiota (Fig. 7C) revealed that the gut: 5-methoxytryptamine ratio and the hippocampus: xanthurenic-acid ratio were significantly correlated with behavioral indicators; the hippocampus: 5-methoxytryptamine ratio was associated with different time points and TST struggle times; and the gut: Indole-3-ethanol ratio was correlated with movement distance, immobility time, and FST struggle time. Biochemical indicator correlations revealed that hippocampus: 5-methoxytryptamine and gut: Indole-3-ethanol were significantly associated with serum: IL-1β, serum: CALP, VEGF, HIF-2, CORT, and gut: TNF-α; hippocampus: xanthurenic-acid correlated with serum: IL-1β, serum: CALP, SOD, and CORT; and gut: 5-Methoxytryptamine was linked to serum: IL-1β, serum: CALP, SOD, HIF-2, CORT, and gut: TNF-α. Additionally, gut: 5-methoxytryptamine, hippocampus: xanthurenic-acid, and gut: Indole-3-ethanol were significantly correlated with the gut microbiota taxa *f-Porphyromonadaceae*, *f-Staphylococcaceae*, and *f-Corynebacteriaceae*, whereas hippocampus:5-methoxytryptamine was only significantly correlated with *f-Corynebacteriaceae.*

### 3.10 Validation of Hippocampal Function and Neurotransmitter Levels

**Fig. 8.**
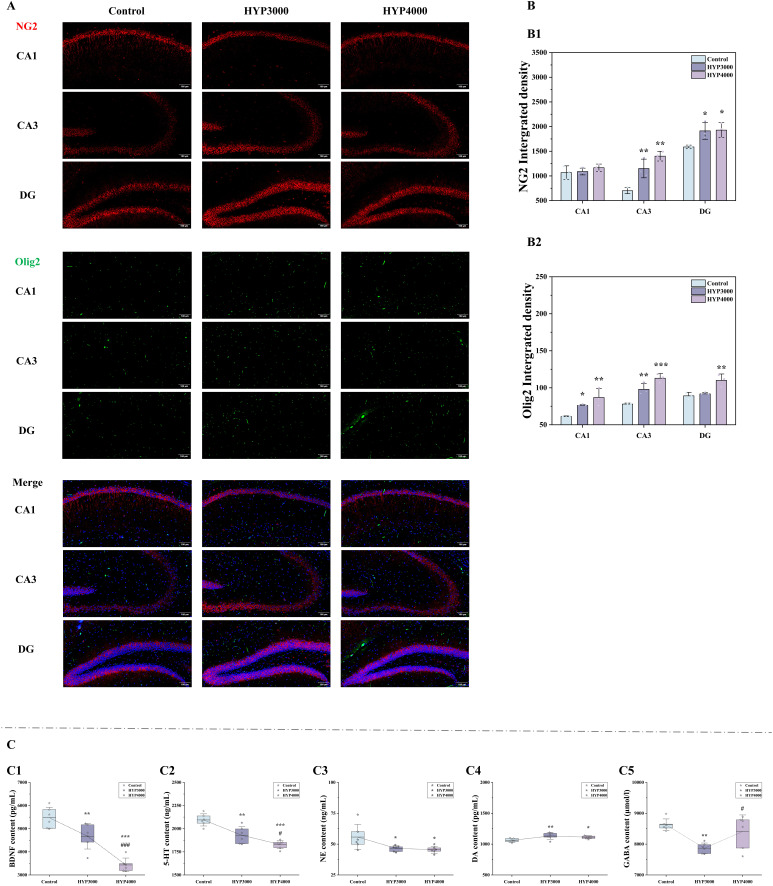
**Effects of hypoxic exposure at different altitudes on hippocampal function in mice**. (A) Immunofluorescence images of neuron–glial antigen 2 (NG2), oligodendrocyte transcription factor 2 (Olig2) and Merge. Scale bar = 100 µm. (B) Fluorescence density (sample size n = 3), (B1,B2) are as follows: fluorescence density of NG2, Olig2. (C) Neurotransmitter levels (sample size n = 6), (C1–C5) are as follows: brain-derived neurotrophic factor (BDNF), 5-hydroxytryptamine (5-HT), norepinephrine (NE), dopamine (DA), gamma-aminobutyric acid (GABA). The data are presented as the means ± SDs. Statistical significance is denoted as follows: *p < 0.05, **p < 0.01, and ***p < 0.001 indicate differences vs. the control group; #*p* < 0.05 and ###*p* < 0.001 indicate differences vs. the HYP3000 group.

Immunofluorescence staining was used to determine the effect of hypoxia on oligodendrocyte precursor cells in each subregion of the mouse hippocampus (Fig. 8A). Compared with that of the control group (Fig. 8B), the fluorescence density of NG2 was greater in the hippocampal CA1 (HYP3000: 2.01%; HYP4000: 9.64%), CA3 (HYP3000: 64.49%, *p* < 0.01; HYP4000: 100.22%, *p* < 0.01), and DG (HYP3000: 20.50%, *p* < 0.05; HYP4000: 21.49%, *p* < 0.05) regions. The fluorescence density of Olig2 also increased in the hippocampal CA1 (HYP3000: 24.53%, *p* < 0.05; HYP4000: 42.00%, *p* < 0.01), CA3 (HYP3000: 22.53%, *p* < 0.01; HYP4000: 44.78%, *p* < 0.001), and DG (HYP3000: 2.95%; HYP4000: 23.46%, *p* < 0.01) regions in the hypoxia groups. Compared with the HYP3000 group, the HYP4000 group presented increased NG2 and Olig2 levels in the CA1 (NG2: 7.09%; Olig2: 14.03%), CA3 (NG2: 21.73%; Olig2: 15.33%, *p* < 0.05), and DG (NG2: 0.82%; Olig2: 19.92%, *p* < 0.01) regions.

Neurotransmitter levels in the mouse cerebral cortex were assessed, and the results (Fig. 8C) revealed that compared with those in the control group, the DA levels in the hypoxia groups were significantly greater (HYP3000: 6.65%, *p* < 0.01; HYP4000: 4.80%, *p* < 0.05). GABA (HYP3000: –8.76%, *p* < 0.01; HYP4000: –2.50%), BDNF (HYP3000: –14.64%, *p* < 0.01; HYP4000: –37.29%, *p* < 0.001), 5-HT (HYP3000: –7.91%, *p* < 0.01; HYP4000: –12.68%, *p* < 0.001), and NE (HYP3000: –16.64%, *p* < 0.05; HYP4000: –18.38%, *p* < 0.05) levels significantly decreased. Compared with the HYP3000 group, the HYP4000 group presented significant increases in GABA (6.86%, *p* < 0.05) and decreases in DA (–1.73%), BDNF (–26.54%, *p* < 0.001), 5-HT (–5.18%, *p* < 0.05), and NE (–2.32%).

## 4. Discussion

Numerous clinical and epidemiological studies have confirmed that high-altitude residence is closely associated with an increased risk of depression. A systematic review and meta-analysis encompassing more than 40,000 participants from 4 countries reported that the prevalence of depressive symptoms in high-altitude (≥1500 m) populations reaches 17.9%, with a striking 28.7% among Chinese high-altitude residents alone [8]. Specifically, a study of 24,141 Tibetans on the Qinghai‒Tibet Plateau reported that 52.3% had depressive symptoms and that 28.6% met the depression diagnostic criteria, significantly exceeding the national average [29], while similar trends have been observed globally, such as higher suicide rates (a severe consequence of depression) in Ecuadorian cantons above 2500 m than in low-altitude regions and increased depressive scores among medical interns stationed at high altitudes [12]. As a widespread nonbiotic environmental stressor, high-altitude hypoxia poses increasing neuropsychiatric risks to human populations amid expanding high-altitude activities [30, 31], critically threatening the well-being of individuals in such environments, and epidemiological evidence further indicates that the prevalence of depression in high-altitude regions is substantially greater than that in low-altitude areas [32]. Nevertheless, there is a lack of systematic and comprehensive investigations into the underlying mechanisms of hypoxia-induced depressive-like behaviors, particularly regarding the involvement and key molecular components of the gut‒brain axis regulatory pathway. Consequently, this study systematically delineated the toxicological cascade of acute hypoxia-induced depressive-like behaviors in mice, highlighting the gut microbiota-tryptophan metabolism axis as a critical mediator of “ecological perturbation-to-neurotoxicity” translation and establishing a clear intensity‒toxicity relationship between hypoxia intensity and toxicity endpoints—findings that not only deepen our understanding of the neurotoxic mechanisms induced by hypoxic exposure but also provide practical translational medical value for environmental health risk assessment in high-altitude regions.

### 4.1 Intensity-Dependent Relationships: Hypoxia Intensity as a Driver of the Toxicity Gradient

First, behavioral tests, including the OFT, TST, and FST, were conducted to evaluate behavioral alterations in mice exposed to a hypoxic environment, with hypoxic groups exhibiting depressive-like behaviors compared with the normoxic control group. Specifically, in the OFT, the HYP4000 group presented 38.69% increases in immobility time (*p* < 0.001) and 60.72% and 32.66% decreases in central dwell time and total movement distance (both *p* < 0.001), whereas in the TST and FST, struggling time was significantly prolonged by 41.23% and 37.58%, respectively (both *p* < 0.01). These findings are consistent with epidemiological evidence that “the prevalence of depressive symptoms (17.9%) is significantly greater among individuals residing in high-altitude regions than among those in low-altitude areas (1.8%–14%)” [7]. In support of the core ecotoxicological principle of an intensity‒toxicity relationship, the HYP4000 group displayed more severe depressive-like behaviors than did the HYP3000 group, with significant decreases in movement distance (–13.21%), center movement distance (–17.23%), and center residence time (–43.84%) and significant increases in immobility time (10.95%), TST-struggle time (19.37%), and FST-struggle time (19.08%). These behavioral observations were corroborated by consistent intensity-dependent patterns in gut microbiota composition, tryptophan pathway metabolites, and tissue damage severity: compared with the HYP3000 group, the HYP4000 group presented significantly greater abundances of the proinflammatory bacterial families *f-Staphylococcaceae* and *f-Corynebacteriaceae* [33, 34], significantly lower colonic levels of the neuroprotective metabolite 5-methoxytryptamine, significantly higher hippocampal levels of the neurotoxic metabolite xanthurenic acid [35, 36], and more severe hippocampal CA3 region cell disorganization and colonic epithelial necrosis—collectively indicating that hypoxia acts as a key environmental trigger for high-altitude depressive symptoms with intensity-dependent effects. Notably, compared with the HYP3000 group, the HYP4000 group presented a paradoxical reduction in the levels of some inflammatory markers, suggesting that the body’s anti-inflammatory capacity may not have been adequately restored and that 4000 m may represent a critical threshold near which immune system collapse initiates [37]. The changes in TNF-α, IL-6, and IL-10 levels were generally consistent between systemic inflammation and local intestinal inflammation. However, there were certain differences in the changes in LPS and IL-1β. Regarding the phenomenon that some pro-inflammatory markers decreased under high hypoxic intensity, the “immunocompensatory dysfunction” proposed in this study is only a preliminary hypothesis. This phenomenon may be related to immune cell exhaustion and anti-inflammatory pathway activation induced by hypoxia [38, 39].

It is important to contextualize the behavioral phenotype of hypoxic mice with their comprehensive behavioral profile and the pathological background of high-altitude hypoxia, as increased struggle time and decreased immobility in the TST/FST—conventionally interpreted as antidepressant-like effects in standard depression models—do not reflect such effects here. In classic depression models (e.g., chronic unpredictable stress model), mice exhibit decreased struggle time [40], while in anxiety models, mice show increased struggle time accompanied by stereotyped behaviors [41], an essential distinction from the “functional struggle” induced by antidepressant drugs. The increased struggle time in the TST and FST observed in this study is neither a traditionally recognized “antidepressant-like effect” nor a pure anxiety phenotype [42]. Instead, it reflects impaired stress adaptation caused by the collapse of the central stress regulatory network under hypoxia-induced comorbid depression-anxiety conditions. Unlike pharmacologically induced antidepressant responses (e.g., selective serotonin reuptake inhibitors that increase synaptic 5-HT availability), hypoxic mice simultaneously displayed reduced center movement distance (–32.66% in HYP4000), shortened center residence time (–60.72% in HYP4000), and decreased total movement distance (–25.05% in HYP4000) in the OFT—core indicators of anxiety-like and anhedonic behaviors, which are hallmark features of depressive-like phenotypes. Instead, the increased struggle time in the TST/FST under hypoxia reflects impaired stress tolerance and dysregulated coping responses, supported by concurrent HPA axis hypofunction (64.92% reduction in CRH in HYP4000) and hippocampal CA3 region disorganization (loose cell arrangement, widened intercellular spaces) that disrupts the neural circuitry mediating stress adaptation. These preclinical findings align with population-based evidence [12], collectively demonstrating that chronic hypobaric hypoxia is a key driver of depressive phenotypes. For high-altitude residents, long-term oxygen deprivation not only triggers systemic inflammation (which is correlated with elevated C-reactive protein levels) and HPA axis dysfunction but also disrupts the gut microbiota balance and tryptophan metabolism—the core mechanisms identified in our mouse model. Additionally, age-specific vulnerabilities exist: middle-aged and elderly individuals are more susceptible at 500~2000 m, whereas young people face greater risks at extreme altitudes (>4000 m) because of incomplete physiological adaptation. This age heterogeneity underscores the need for targeted mental health interventions in high-altitude populations, particularly young migrants and military personnel experiencing acute hypoxic exposure [43].

However, a critical distinction must be made between anxiety-like and depression-like phenotypes induced by hypoxia, as they involve distinct neural circuits yet share common pathogenic pathways. OFT-detected reductions in central zone exploration are unequivocally associated with anxiety, reflecting hyperactivation of the amygdala‒hippocampal fear circuit. Our data align with previous findings that high-altitude hypoxia triggers anxiety-like behaviors via HPA axis overactivation (evidenced by Arginine Vasopressin/Glucocorticoid Receptor (AVP/GR) upregulation in the hypothalamic paraventricular nucleus) and neuroinflammatory responses. In contrast, TST/FST behavioral alterations reflect depression-related dysregulation of stress coping and are correlated with disrupted serotonergic signaling in the prefrontal cortex‒hippocampal pathway. Notably, anxiety and depression often co-occur under hypoxic stress, which is supported by our multidimensional data. Serum levels of IL-1β and gut TNF-α (key proinflammatory factors) were positively correlated with both OFT central zone avoidance and TST/FST struggle time (Fig. 7C), indicating shared inflammatory mechanisms. Moreover, tryptophan metabolism dysregulation (e.g., reduced 5-methoxytryptamine and elevated xanthurenic acid levels) simultaneously impacts anxiety (via amygdala 5-HT receptors) and depression (via hippocampal glutamatergic transmission). Furthermore, HPA axis dysfunction (CRH/CORT reduction) disrupts the coordination of stress responses, promoting both anxiety-related risk aversion and depression-related coping impairment. These findings support the hypothesis that hypoxia contributes to a complex negative emotional state encompassing both anxiety and depression rather than a single emotional disorder.

### 4.2 Gut Microbiota‒Tryptophan Metabolism Axis: The Core Toxicological Cascade

Our results identify the gut microbiota-tryptophan metabolism axis as the central pathway that translates hypoxic environmental stress into neurotoxicity. On the basis of our previous findings, hypoxia disrupts the composition of the gut microbiota [44, 45], which serves as a central mediator in brain‒gut axis communication [12]. Therefore, we investigated alterations in the intestinal microbial community. 16S rRNA sequencing revealed that microbial diversity in the hypoxic mouse groups was significantly reduced, as indicated by decreased Shannon, Simpson, and Chao1 indices (Shannon index: *p* < 0.01 in the HYP3000 group), a pattern commonly observed in psychiatric conditions such as depression [46]. At the taxonomic level, the proinflammatory bacterial families [47] *f-Staphylococcaceae* and *f-Corynebacteriaceae* were markedly enriched (*f-Staphylococcaceae* increased 14.6-fold in the HYP4000 group, *p* < 0.001), whereas the SCFA-producing families [48, 49] *f-Lachnospiraceae* and *f-Ruminococcaceae* were significantly depleted (*f-Lachnospiraceae* decreased by 64.1% in the HYP4000 group). SCFAs are known to support intestinal barrier integrity, suppress inflammation, and modulate neurotransmitter synthesis [50]; their reduction may therefore exacerbate both intestinal dysfunction and depressive phenotypes. Fecal and hippocampal metabolomic analyses revealed that tryptophan metabolism was a shared dysregulated pathway in both compartments under hypoxia. In the gut, all 11 detected tryptophan metabolites were downregulated in the HYP3000 group (e.g., 5-methoxytryptamine decreased by 62.56%, *p* < 0.001), whereas the HYP4000 group exhibited a “bidirectional regulation” pattern (5-methoxytryptamine decreased by 74.21%, serotonin increased by 25.86%, both *p* < 0.01). In the hippocampus of hypoxic mice, the level of serotonin, an antidepressant neurotransmitter [51], was reduced (by 33.41% in the HYP3000 group; *p* < 0.001), whereas the level of xanthurenic acid, a neurotoxic compound implicated in neuronal injury [52], was elevated (by 67.34% in the HYP4000 group; *p* < 0.001). *f-Staphylococcaceae* and *f-Corynebacteriaceae* are positively correlated with tryptophan-derived neurotoxins and negatively correlated with neuroprotectants, indicating that gut microbiota dysbiosis may directly contribute to metabolic toxicity. Additionally, key metabolites, such as intestinal 5-methoxytryptamine and hippocampal xanthurenic acid, are significantly associated with behavioral parameters and inflammatory markers. These findings suggest that hypoxia can regulate tryptophan metabolism by altering the gut microbiota and subsequently transmitting metabolic toxicity through the gut‒brain axis to affect hippocampal function. Gut-derived metabolites and inflammatory factors induce inflammatory responses, oxidative stress, and HPA axis dysregulation in the body; trigger structural damage and functional deficits in the hippocampus; and ultimately lead to depressive-like behaviors. The integration of multidimensional data further validated the interpretation of the depressive-like phenotype. In addition to the TST/FST, hypoxic mice presented reduced exploratory behavior (OFT center parameters) and disrupted neurotransmitter balance (cerebral cortex 5-HT reduction by 12.68%, BDNF reduction by 37.29% in HYP4000), which are independently linked to depression pathogenesis [51]. Notably, Spearman correlation analysis (Fig. 7C) revealed that the TST/FST duration was positively correlated with the levels of neurotoxic metabolites (hippocampal xanthurenic acid) and proinflammatory factors (serum: IL-1β and gut: TNF-α) and negatively correlated with the levels of neuroprotective metabolites (gut: 5-methoxytryptamine). These findings indicate that the increased struggle time is driven by neurotoxicity and inflammation, not antidepressant adaptation. Similar observations were reported in a hypobaric hypoxia mouse model by Bakshi and Mishra (2025) [16], where increased FST time was associated with intestinal barrier injury and hippocampal microglial activation and was reversed by fecal microbiota transplantation, which normalized tryptophan metabolism, confirming the causal link between hypoxia-induced gut–brain axis disruption and the observed behavioral phenotype. Notably, the changes in key metabolites of the tryptophan metabolic pathway are not entirely consistent between the intestine and hippocampus, and some metabolites exhibit opposite regulatory trends (e.g., serotonin). The putative reasons may be related to blood-brain barrier (BBB) transport dysfunction, activation of competitive metabolic pathways, or tissue-specific regulation. As a critical structure separating the peripheral circulation from the CNS, the BBB regulates the transmembrane transport of metabolites through specific transporters and tight junctions, and its dysfunction is one of the core links leading to the imbalance of tryptophan metabolites between the periphery and the central nervous system [53]. Research has shown that cerebral hypoxia can upregulate the expression of amino acid transporters solute carrier family 7 member 5/solute carrier family 3 member 2 (SLC7A5/SLC3A2) heterodimeric amino acid transporter [54], which may induce differences in the supply of tryptophan precursors and thus lead to variations in the levels of related metabolites. The tryptophan metabolic pathway consists of two branches: the serotonin pathway and the kynurenine pathway. The serotonin pathway can produce neuroprotective substances such as serotonin and melatonin. There are complex interactions between the two pathways, and the scientific community currently holds the hypothesis that the imbalance of these two metabolic pathways can induce depression [55]. Therefore, hypoxia may cause the dysregulation of both pathways, resulting in differences between different tissues.

These findings address a critical knowledge gap regarding key metabolic nodes within the “hypoxia–gut microbiota–depression” axis. Collectively, our results suggest that gut microbiota dysbiosis acts as an initiating factor in hypoxia-induced depression, driving aberrant tryptophan metabolism and mediating hippocampal dysfunction via the gut‒brain axis, which is the core mechanism elucidated in this study.

As previously discussed, aberrant hippocampal neural function is the terminal neurotoxic effector pathway of hypoxia-induced depressive-like behaviors, which are intricately linked to the aforementioned gut‒brain axis mechanism, collectively forming a comprehensive hypoxia‒related network that is correlated with the regulatory network of toxicity. Oligodendrocytes are increasingly recognized as key players in neuroimmune modulation under environmental stress [56]; thus, we examined oligodendrocyte dynamics in the hippocampus. Immunofluorescence analysis revealed a significant increase in the fluorescence density of the oligodendrocyte precursor cell (OPC) markers NG2 and Olig2 in the hippocampal CA3 and DG regions of the hypoxic groups; specifically, NG2 expression in the HYP4000 group increased by 100.22% (*p* < 0.01). The increased number of NG2/Olig2-positive OPCs in hypoxic mice cannot be simply interpreted as toxic injury; instead, it represents a nuanced interplay between the injury response and compensatory repair, as OPCs serve as the primary “repair reserve” of the central nervous system, and their rapid proliferation following hypoxic insult is a conserved adaptive response to tissue damage—serving to replenish the oligodendrocyte pool and promote myelin regeneration. These results are consistent with previous studies [57, 58] showing that chronic cerebral hypoperfusion induces OPC proliferation within 1 month, which contributes to the restoration of mature oligodendrocyte populations, although the effectiveness of this repair depends on the ability of proliferated OPCs to differentiate into functional myelin-forming oligodendrocytes and whether such differentiation into mature oligodendrocytes for myelin repair actually occurs remains to be validated.

Additional neurotransmitter profiling revealed that the levels of antidepressant-associated neurotransmitters, including GABA, BDNF, 5-HT, and NE, in the cerebral cortex were significantly reduced under hypoxic conditions (BDNF levels in the HYP4000 group decreased by 37.29%, *p* < 0.001), with only DA showing a modest increase. These findings align with the reduction in hippocampal serotonin caused by disrupted tryptophan metabolism, further confirming that hypoxia exacerbates depressive symptoms via the suppression of neurotransmitter synthesis—an important neurotoxic mechanism—and verifying the “gut‒hippocampus metabolic toxic axis” (ecological-health translational value of gut metabolism to brain toxicity). Collectively, the results reveal the following mechanistic cascade: hypoxic exposure → intestinal dysbiosis (increased proinflammatory bacteria and decreased beneficial microbiota) → intestinal barrier impairment and aberrant tryptophan metabolism → systemic inflammation and oxidative stress activation, along with HPA axis desensitization → hippocampal tryptophan metabolic disturbances (accumulation of xanthurenic acid and reduced serotonin) and neurotransmitter imbalance → hippocampal structural damage and functional deficits → manifestation of depressive behaviors. This pathophysiological pathway highlights the differential effects of varying hypoxia intensities and provides well-defined molecular targets and an experimental foundation for the prevention and intervention of high-altitude depression. This pathway also highlights the hypoxia intensity-dependent gradient and provides well-defined toxicological targets for preventing high-altitude depression.

Although new findings have been reported, this study has some limitations. (1) Limitations of the animal models: The present study utilized only male KM mice aged 5–6 weeks and did not include female subjects or other mouse strains, such as C57BL/6. Sex differences may significantly influence susceptibility to depression; for example, females are generally more sensitive to hypoxia-induced stress, and interstrain variations exist in both the composition of the gut microbiota and hypoxia tolerance. Therefore, the generalizability of the findings to other sexes or genetic backgrounds may be limited. (2) Duration of hypoxic exposure: This study focused exclusively on acute hypoxia over a 7-day period and did not examine chronic hypoxic exposure for more than one month. Given that most individuals residing at high altitudes experience long-term hypoxic conditions, chronic hypoxia may induce adaptive alterations in the gut microbiota and metabolic profiles that differ mechanistically from those observed under acute exposure. Additionally, the study did not investigate recovery following cessation of hypoxia, thus precluding evaluation of the reversibility of the observed effects. (3) Causal Relationships Not Established: Although correlations were identified among the gut microbiota, tryptophan metabolism, and depressive-like behaviors, the causal relationships remain unverified. The absence of interventions such as fecal microbiota transplantation (e.g., transferring microbiota from hypoxic mice to germ-free recipients) or direct metabolite supplementation (e.g., administration of 5-methoxytryptamine) limits the ability to determine whether microbial or metabolic changes directly contribute to depressive phenotypes. Although both KEGG functional prediction of gut microbiota and untargeted metabolomics indicate the critical role of the tryptophan metabolic pathway, the functional prediction of gut microbiota is a speculative conclusion, and the regulatory effect of key gut microbiota on the tryptophan metabolic pathway lacks further verification of causal relationships. (4) Scope of Measured Outcomes: The behavioral assessments were restricted to depressive-like behaviors and did not extend to cognitive functions, such as spatial learning and memory (e.g., via the Morris water maze). The hippocampal analyses focused solely on OPCs without evaluating other critical neurobiological markers, such as neuronal apoptosis or microglial activation. Furthermore, the functional potential of the gut microbiota was inferred through KEGG pathway prediction rather than direct measurement of key metabolites such as SCFAs or bile acids, which may have led to an underestimation of functional microbial changes. (5) Clinical translatability: The findings derived from animal models require validation in human populations. To date, clinical data on the gut microbiota composition and tryptophan metabolite levels in individuals with depression living at high altitudes are lacking. Consequently, whether the mechanisms observed in this murine model are applicable to humans remains uncertain.

Therefore, on the basis of the innovations and limitations of this study, further research is still needed in the following aspects. (1) Expanding animal models and modeling conditions: Female mice, more mouse strains and nonhuman primates should be used to investigate potential sex- and species-specific differences. Chronic hypoxia groups with exposure durations of one month or longer, as well as reoxygenation recovery groups after hypoxia cessation, should be established to evaluate the long-term effects and reversibility of hypoxia exposure. (2) Validation of causal relationships: A key limitation of this study is its observational and correlational design, as no intervention experiments (e.g., fecal microbiota transplantation (FMT), antibiotic-induced gut microbiota depletion, or supplementation with key taxa/metabolites) were performed to verify causal relationships between changes in the gut microbiota and emotional phenotypes. While multiomics correlation analyses support a potential regulatory network, causality cannot be definitively inferred from associative data alone, as noted in prior microbiota‒gut‒brain axis studies. Future studies should use intervention approaches (e.g., FMT) to validate whether targeting the gut microbiota or tryptophan metabolism can reverse hypoxia-induced emotional and neurobiological alterations, thereby confirming causal links. (3) Clinical translation: Observational cohort studies in high-altitude populations should be conducted to verify the translational relevance of findings from animal models and targeted clinical intervention trials for high-risk populations in high-altitude areas should be designed and implemented to evaluate the intervention effects of probiotic supplementation or pharmacological regulation of the tryptophan metabolic pathway. (4) Limitation of sucrose preference test (SPT) exclusion and future optimization: This study did not incorporate the SPT because of inconsistent preliminary results, which were attributed to evaporation of the sucrose solution and inherent spatial preferences in mice. As a result, anhedonia could not be directly assessed, representing a key limitation of the present work. Future studies may address this limitation by optimizing the experimental protocol through the use of sealed containers, standardized placement of drinking bottles, and adequate preadaptation of the mice to the testing environment. (5) This study did not assess OPC differentiation status (e.g., the mature oligodendrocyte marker myelin basic protein (MBP) or Cyclin-dependent kinase inhibitor 1 (CC1)) or perform *in vivo* tracking of OPC fate. Future studies should combine lineage tracing and differentiation marker detection to clarify whether hypoxic OPCs can successfully mature into functional oligodendrocytes. Additionally, intervention experiments (e.g., growth hormone supplementation or CRH signaling modulation) could be used to verify whether promoting OPC differentiation can reverse hypoxia-induced hippocampal dysfunction, thereby validating the causal role of insufficient OPC repair in the observed phenotypes.

## 5. Conclusions

This study revealed strong correlations between acute hypoxia, gut microbiota imbalance, tryptophan metabolism disruption, inflammatory responses, HPA axis dysfunction, and a complex negative emotional state encompassing anxiety- and depression-like behaviors. These findings highlight a potential regulatory network centered on the gut microbiota‒tryptophan metabolism axis, providing a foundation for future intervention studies to validate causal relationships and explore therapeutic targets for hypoxia-associated emotional disturbances. Our findings provide a toxicological framework for understanding high-altitude hypoxia-induced depression and offer translational tools for protecting the health of populations in high-altitude ecosystems.

## Availability of Data and Materials

The authors declare that all the data supporting the findings of this study are available upon reasonable request. Raw read files (SRAs) for 16S rRNA sequencing of gut bacteria were deposited into the National Center for Biotechnology Information Sequence database (BioProject: PRJNA1267953). As these data are related to our upcoming experiments and articles, they will not be accessible until September 1, 2026.
